# Purification and Characterization of Anti-Inflammatory Peptide Fractions from Enzymatic Hydrolysate of Abalone Viscera

**DOI:** 10.3390/foods14223811

**Published:** 2025-11-07

**Authors:** Nan Wu, Ziyi Yang, Chaocheng Wu, Yuan Chen, Zhuhua Chan, Runying Zeng

**Affiliations:** Technology Innovation Center for Exploitation of Marine Biological Resources, Third Institute of Oceanography, Ministry of Natural Resources, Xiamen 361005, China; wunan@tio.org.cn (N.W.);

**Keywords:** abalone viscera, enzymatic peptides, antioxidant activity, anti-inflammatory mechanism, MAPK signaling pathway

## Abstract

Roughly 25% of abalone viscera generated during processing is currently discarded, resulting in substantial protein wastage and environmental contamination. In the present study, abalone viscera served as the raw material; four commercial proteases—papain, bromelain, neutral protease and trypsin—were comparatively evaluated. Among them, the neutral-protease hydrolysate of abalone viscera (AVZH) exhibited the strongest suppression of nitric oxide (NO) release from lipopolysaccharide (LPS)-stimulated RAW264.7 macrophages. Liquid chromatography with tandem mass spectrometry (LC-MS/MS) analysis ultimately led to the identification of 18 novel peptides. Integrating bioinformatic prediction with solid-phase synthesis, two sequences—GYSFTTTAER and IKKPPQDEWGTGL—were further screened and confirmed to possess excellent cytocompatibility and pronounced anti-inflammatory potency. Mechanistic investigations revealed that both peptides dose-dependently attenuated the secretion and mRNA expression of IL-1β, IL-6 and TNF-α and concurrently blocked mitogen-activated protein kinase (MAPK) signaling by down-regulating the phosphorylation of ERK, JNK and p38. These findings demonstrate that abalone viscera represent an abundant reservoir of anti-inflammatory peptides, offering both a theoretical framework and a technological roadmap for the valorisation of marine waste proteins and the development of next-generation natural anti-inflammatory agents.

## 1. Introduction

Chronic inflammation drives many diseases, including cardiovascular and neurodegenerative conditions [1]. This process is characterized by the overproduction of nitric oxide (NO) and pro-inflammatory cytokines like Tumour Necrosis Factor-alpha (TNF-α) and Interleukin-6 (IL-6). Although conventional anti-inflammatory therapies are effective, they are frequently accompanied by adverse side effects [2], necessitating the search for safer natural alternatives. Marine ecosystems account for nearly half of global biodiversity; the extreme conditions of high salinity, elevated pressure and low temperature have driven marine organisms to evolve an extraordinary array of structurally unique and functionally diverse bioactive peptides [3]. Once liberated by enzymatic hydrolysis, these peptides frequently display more potent antibacterial, anti-inflammatory, antihypertensive and immunomodulatory activities than their terrestrial counterparts [4].

Abalone is a high-value aquaculture species with global production exceeding 200,000 t yr^−1^, predominantly concentrated in Asia [5]. Abalone viscera (including digestive and gonadal glands) account for 25% of their total body weight and are rich in protein (approximately 40–50% by dry weight), making them an ideal substrate for enzymatic hydrolysis to produce bioactive peptides with anti-inflammatory and antioxidant properties. However, abalone viscera are discarded as a fishery by-product during processing, resulting in a loss of protein resources and a burden on the environment [6]. Compared with the adductor muscle or polysaccharide fractions, the valorisation of abalone viscera remains largely unexplored despite its favourable profile of essential amino acids and hydrophobic residues linked to bioactive potential [7]. Proteins in abalone viscera have value-added potential as precursors of bioactive peptides. A large amount of evidence shows that abalone contains active compounds such as peptides and polysaccharides with various biological activities, including nutritional and health properties and pharmacological properties [8]. Sensory defects and perceived contamination have historically relegated this by-product to low-value uses. Recent studies nevertheless revealed that organic extracts and proteolytic hydrolysates of abalone viscera possess anti-inflammatory activity, manifested by free-radical scavenging, the suppression of LPS-induced NO and Reactive Oxygen Species (ROS) generation, down-regulation of the nuclear factor kappa B (NF-κB) pathway, and diminished secretion of Interleukin-1 Beta (IL-1β), IL-6 and TNF-α [9]. Nevertheless, targeted enzymatic protocols, anti-inflammatory peptide sequences and their precise mechanisms remain uncharted.

For food and pharmaceutical applications, enzymatic hydrolysis remains the method of choice for producing bioactive peptides owing to its mild conditions, high efficiency, precise controllability and selectivity, and the absence of residual organic solvents or toxic chemicals in the final product. Previous investigations have confirmed the suitability of this approach for recovering bioactive peptides from tilapia processing discards [10]. Critical steps in the process include substrate preparation, judicious enzyme selection, precise control of the degree of hydrolysis, homogenisation, heat inactivation of endogenous enzymes, hydrolysis per se and timely termination of the reaction. Process parameters such as enzyme concentration, pH, incubation time and temperature must likewise be rigorously controlled. Commercially available enzymes commonly employed include alcalase, neutral protease and flavourzyme, whereas animal- and plant-derived preparations include trypsin, pepsin, papain and bromelain [11].

This study systematically compared the efficiency of four proteases—papain, bromelain, neutral protease and trypsin—in generating hydrolysates with concomitant antioxidant and anti-inflammatory activities. AVZH emerged as the most potent inhibitor of NO production in LPS-stimulated RAW264.7 macrophages. Subsequent LC-MS/MS profiling identified 18 novel peptides, two of which—GYSFTTTAER and IKKPPQDEWGTGL—exhibited exceptional anti-inflammatory efficacy. These peptides not only suppressed the secretion of pro-inflammatory cytokines (IL-1β, IL-6 and TNF-α) but also blunted MAPK pathway activation by down-regulating phosphorylated ERK, JNK and p38. For the first time, abalone viscera-derived peptides have been mechanistically linked to MAPK-mediated anti-inflammatory activity, thereby expanding the repertoire of marine-derived bioactives.

## 2. Method

### 2.1. Collection and Preparation of Abalone Viscera

Visceral by-products (digestive gland and gonad) were obtained from the processing line of Tannan Bay Aquatic Products Co., Ltd. (Pingtan, China). The material was thoroughly rinsed with chilled tap water to remove residual salt and impurities, minced under aseptic conditions, lyophilised (−50 °C, 48 h) and finally ground to a fine powder (<250 µm). The powder was vacuum-sealed and stored at −20 °C until use.

### 2.2. Enzymatic Hydrolysis

Hydrolysis conditions (pH, temperature, time) were selected based on preliminary single-factor optimization and previous studies on marine by-product hydrolysis [12]. The enzyme dosage and hydrolysis parameters used in the article were all based on the optimal reaction conditions of the enzyme. Freeze-dried viscera powder was suspended in sterile distilled water at a solid-to-liquid ratio of 1:20 (*w*/*v*). The pH was adjusted to the optimum for each enzyme (papain, 6.5; bromelain, 7.0; neutral protease, 7.5; trypsin, 8.0) using 1 M NaOH or HCl. Four commercial proteases (Sigma-Aldrich) were used at an enzyme-to-substrate ratio of 3000 U/g protein: papain (100,000 U/g), bromelain (100,000 U/g), neutral protease (150,000 U/g), and trypsin (200,000 U/g). The hydrolysis reactions were carried out for 5 h with gentle agitation at the respective optimal temperatures: 60 °C for papain, 50 °C for bromelain and neutral protease, and 37 °C for trypsin Reactions were terminated by heating at 95 °C for 10 min to inactivate the enzymes. After cooling on ice, the mixtures were centrifuged at 10,000× *g* for 20 min (4 °C). The supernatants were collected, lyophilised, reconstituted in ultrapure water and ultrafiltered through a 3 kDa molecular-weight-cut-off membrane to obtain the hydrolysates, which were designated as AVMH (papain hydrolysate of abalone viscera), AVBH (bromelain hydrolysate of abalone viscera), AVZH, and AVYH (trypsin hydrolysate of abalone viscera).

### 2.3. Compositional Analyses

Crude protein was determined by the Kjeldahl method (N × 6.25); total carbohydrates by the phenol-sulphuric acid assay using D-glucose as standard; moisture by gravimetry after drying at 105 °C to constant mass. Peptide content in hydrolysates was quantified by the BCA method at 562 nm and expressed as % of total protein. Degree of hydrolysis (DH) was calculated by formaldehyde titration: liberated amino nitrogen was titrated with 0.1 M NaOH to pH 9.0, and DH (%) was expressed as the ratio of amino nitrogen to total nitrogen.

### 2.4. Molecular-Weight Distribution

The molecular-weight distribution of the hydrolysates was determined by high-performance gel-permeation chromatography (HP-GPC). This technique was selected over SDS-PAGE due to its superior resolution and quantitative accuracy for low-molecular-weight peptides (<5 kDa), which constitute the primary components of enzymatic hydrolysates. An aliquot of each hydrolysate was filtered through a 0.45 µm membrane and analysed on a PL aquagel-OH Mixed-H column (7.5 × 300 mm, 8 µm) using an isocratic elution with 0.1 M NaNO_3_ containing 0.01% NaN_3_ at 1.0 mL min^−1^ and 45 °C. Molecular-weight standards (180–25,000 Da) were used for calibration, and data were processed with GPC software, version A.02.02 (Agilent, Santa Clara, CA, USA).

### 2.5. Antioxidant and Anti-Inflammatory Assays

Antioxidant capacity was evaluated by (i) total antioxidant capacity using the ferric-reducing antioxidant power assay, (ii) DPPH• scavenging, (iii) •OH scavenging and (iv) ABTS+ scavenging. Anti-inflammatory activity was assessed in RAW264.7 murine macrophages stimulated with 1 µg mL^−1^ LPS. In this initial screening phase, the antioxidant and anti-inflammatory activities of the hydrolysates and peptides were evaluated against the LPS-stimulated or blank controls without the inclusion of pharmaceutical positive controls, to directly compare their intrinsic efficacy. Nitrite accumulation in culture medium was measured by the Griess reaction after 24 h treatment with serial dilutions of each hydrolysate (100–300 µg mL^−1^).

### 2.6. LC-MS/MS Profiling and Peptide Identification

Peptide samples were desalted using C18 columns, dried by centrifugal evaporation, and stored at −20 °C. LC-MS/MS analysis was performed on a Q Exactive HF coupled with an UltiMate 3000 nanoLC system. Samples were separated on a C18 analytical column (75 μm × 25 cm, 1.9 μm, 120 Å) with a gradient of solvent A (0.1% formic acid, 3% DMSO) and solvent B (0.1% formic acid, 3% DMSO, 80% acetonitrile) at 300 nL/min. Data were acquired in DDA mode with one full MS scan (R = 60,000, AGC = 3 × 10^6^, IT = 25 ms, *m*/*z* 350–1500) followed by 20 MS/MS scans (R = 15,000, AGC = 1 × 10^5^, IT = 50 ms) using HCD at 27 NCE. Data were processed with MaxQuant (v1.6.6, Andromeda) against the Haliotis discus hannai protein database, with oxidation (M) and acetylation (protein N-term) as variable modifications and carbamidomethylation (C) as a fixed modification. Identifications were filtered at 1% FDR.

Bioinformatic analyses of identified peptides included physicochemical property calculation (PEPLab-DMPep, Innovagen PepCalc), novelty assessment (BIOPEP-UWM, BioPepDB, EROP-Moscow), bioactivity probability ranking (PeptideRanker), anti-inflammatory prediction (AIPpred, PreAIP), and ADMET/cosmetic safety evaluation (admetSAR 3.0). Peptide sequences were visualized using PepDraw.

### 2.7. Bioinformatic Analysis and Peptide Selection

Peptide sequences were retrieved from the LC-MS/MS datasets. Physicochemical parameters (MW, pI, hydrophobicity) were computed with the PEP Lab-DMPep server. Solubility and extinction coefficients were predicted using Innovagen PepCalc. Novelty was cross-checked against BIOPEP-UWM [13], BioPepDB [14] and EROP-Moscow [15]. Potential anti-inflammatory activity was ranked with PeptideRanker [16] and further predicted via AIPpred [17] and PreAIP [18].

### 2.8. Solid-Phase Synthesis of Anti-Inflammatory Peptides

Five high-ranking peptides (GYSFTTTAER, IKKPPQDEWGTGL, QEYDESGPSIVHR, ELTALNEKYP and PGPAGPLG) were chemically synthesised by Fmoc solid-phase chemistry (≥95% purity) by Sangon Biotech (Shanghai, China). Purity and identity were confirmed by RP-HPLC and ESI-MS.

### 2.9. Cell Culture and Treatment

RAW264.7 macrophages (Cell Bank, Chinese Academy of Sciences, Beijing, China) were cultured in DMEM high-glucose medium supplemented with 10% (*v*/*v*) foetal bovine serum and 1% penicillin-streptomycin at 37 °C in a humidified 5% CO_2_ atmosphere. Cells were seeded at 5 × 10^4^ cells cm^−2^ and treated with indicated concentrations of hydrolysates or synthetic peptides for 24 h prior to further analysis.

### 2.10. Western Blotting

After treatment, cells were lysed with RIPA buffer containing protease and phosphatase inhibitors. Protein concentration was determined by BCA assay. Equal amounts (30 µg lane^−1^) were resolved by 12% SDS-PAGE, transferred to PVDF membranes and blocked with 5% non-fat milk. Membranes were incubated overnight at 4 °C with primary antibodies against phospho-ERK1/2, ERK1/2, phospho-JNK, JNK, phospho-p38 and p38 (Aifang Biotechnology, Changsha, China). After washing, horseradish-peroxidase-conjugated secondary antibodies were applied for 1 h at room temperature. Signals were developed with ECL reagent and captured by a gel-documentation system.

### 2.11. Quantitative Real-Time PCR (qPCR)

Total RNA was extracted using TRIzol reagent and quantified on a NanoDrop 2000. First-strand cDNA was synthesised from 1 µg RNA using a reverse-transcription kit. qPCR was performed on a LightCycler 96 system using SYBR Green Master Mix and gene-specific primers (Table 1). Cycling conditions: 95 °C for 30 s, followed by 40 cycles of 95 °C for 5 s and 60 °C for 30 s. Relative gene expression was calculated using the 2^−ΔΔCt^ method, normalised to GAPDH.

### 2.12. Statistical Analysis

The normality of the data was assessed using the Shapiro–Wilk test before performing one-way ANOVA. All experiments were carried out in triplicate and data are expressed as mean ± standard deviation (SD). One-way ANOVA followed by Tukey’s multiple-comparison test was conducted using SPSS 25.0. Differences were considered statistically significant at *p* < 0.05.

## 3. Results

### 3.1. Compositional Characterisation of Abalone Visceral Hydrolysates

**Figure 1:**
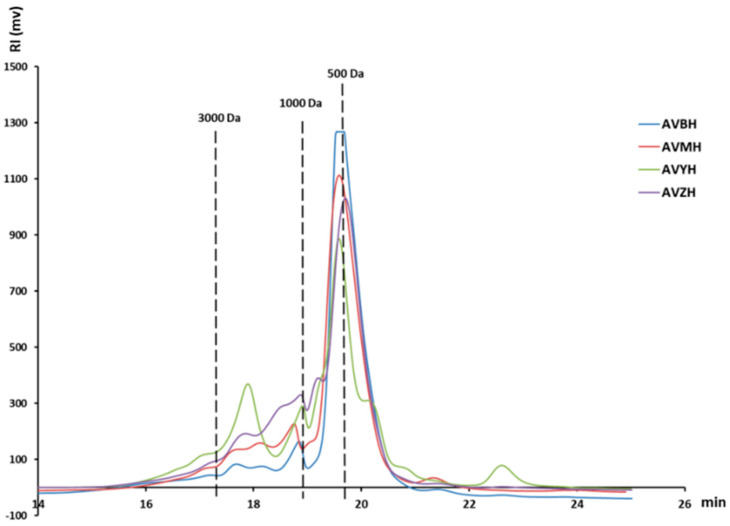
Molecular weight distribution of four types of abalone visceral enzymatic hydrolysates (AVMH, AVBH, AVYH, AVZH).

The dried abalone viscera powder contained 45.09 ± 2.48% protein, 8.54 ± 2.96% total sugars, and 7.99 ± 0.98% moisture. Hydrolysis with different proteases yielded four distinct hydrolysates: AVMH, AVBH, AVZH, and AVYH. The crude hydrolysates showed no significant changes in total sugar content, while all samples exhibited a slight increase in protein levels (Table 2). Proximate and biochemical analyses revealed pronounced differences among the treatments (Table S1). AVZH exhibited the highest peptide yield (68.38 ± 3.87% of total protein) and degree of hydrolysis (DH = 12.44 ± 0.76%), underscoring the superior proteolytic efficiency of neutral protease under the conditions employed. Amino-acid profiling demonstrated a marked enrichment in hydrophobic residues—particularly valine and leucine—after enzymatic cleavage (Table S2). Gel-permeation chromatography further indicated that 500–3000 Da peptides constituted the predominant fraction across all hydrolysates (51.63–73.46%; Figure 1, Table S3). Collectively, these data establish AVZH as the most promising hydrolysate in terms of both peptide abundance and desirable molecular-weight distribution, providing an ideal matrix for subsequent antioxidant and anti-inflammatory evaluations.

### 3.2. Antioxidant Capacity of the Enzymatic Hydrolysates

**Figure 2:**
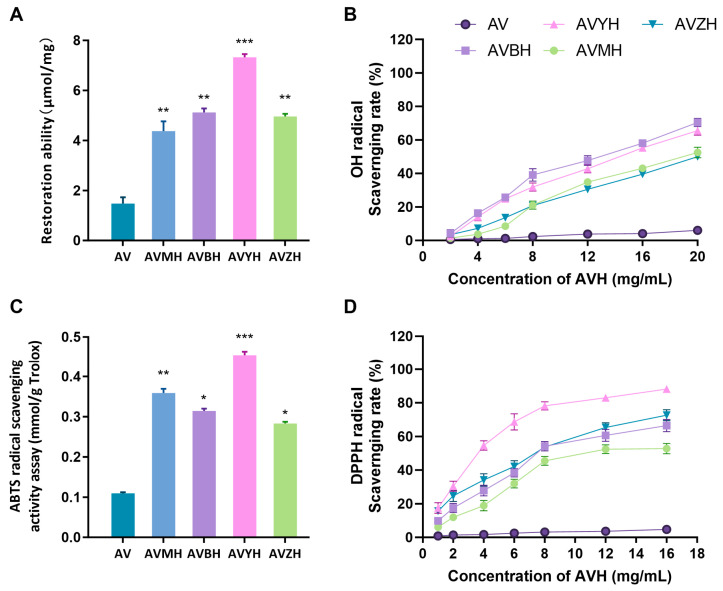
Antioxidant activities of abalone visceral hydrolysates. (**A**) Restoration ability, (**B**) hydroxyl radical scavenging rate, (**C**) ABTS+ radical scavenging activity, and (**D**) DPPH radical scavenging rate of different enzymatic hydrolysates (AV, AVMH, AVBH, AVYH and AVZH). Values represent mean ± SD (*n* = 4); * *p* < 0.05, ** *p* < 0.01, *** *p* < 0.001 vs. AV group.

The in vitro antioxidant profile of the four hydrolysates was systematically evaluated through multiple complementary assays (Figure 2). Among them, AVYH delivered the highest total antioxidant capacity, the strongest DPPH• scavenging activity and the greatest ABTS+ quenching potential. Conversely, AVBH exhibited the most potent hydroxyl-radical scavenging capacity. Across all preparations, antioxidant efficacy exhibited a positive correlation with the relative abundance of low-molecular-weight peptides (<500 Da) and with the proportion of hydrophobic amino-acid residues, indicating that these small, hydrophobic peptides are the principal contributors to radical-scavenging activity. These results provide a rational basis for prioritising hydrolysates with superior antioxidant attributes for downstream peptide isolation and functional validation.

### 3.3. Effects of Hydrolysates on RAW264.7 Macrophage Viability and NO Production

**Figure 3:**
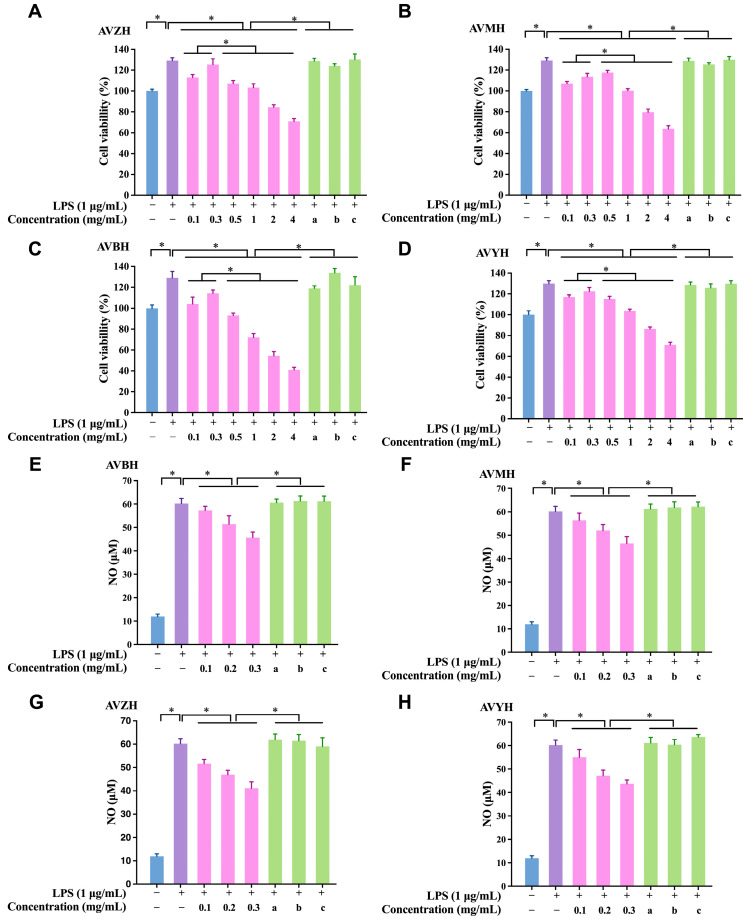
Effects of abalone visceral hydrolysates on RAW264.7 macrophage viability and nitric-oxide (NO) production. (**A**–**D**) Cell viability after 24 h exposure to increasing concentrations (0–300 µg mL^−1^) of AVZH, AVMH, AVBH and AVYH in the presence of 1 µg mL^−1^ LPS. Data are expressed as % of the LPS-only control. (**E**–**H**) Corresponding NO release (µM) under identical conditions. Values represent mean ± SD (*n* = 4); * *p* < 0.05. (a) Cells treated with non-enzymatically hydrolyzed abalone viscera; (b) Cells treated with inactivated protease; (c) Cells treated with abalone viscera enzymatic hydrolysate for 0 h.

Within the concentration window of 100–300 µg mL^−1^, all four hydrolysates markedly promoted RAW264.7 cell proliferation in a concentration-dependent manner (Figure 3A–D). At 300 µg mL^−1^, AVZH evinced the most pronounced proliferative effect, elevating viability by 25.37 ± 1.18% relative to the untreated control. In the LPS-stimulated inflammatory model, NO secretion was substantially augmented in vehicle-treated cells. Each hydrolysate dose-dependently attenuated this response (Figure 3E–H). Again, AVZH displayed the strongest inhibitory potency, suppressing NO release by 31.64 ± 2.05% at 300 µg mL^−1^. These findings demonstrate that abalone visceral hydrolysates not only exhibit excellent cytocompatibility but also possess robust anti-inflammatory activity, providing a solid foundation for subsequent peptide identification and mechanistic elucidation.

### 3.4. Identification, In Silico Screening and Chemical Validation of Anti-Inflammatory Peptides from AVZH

**Figure 4:**
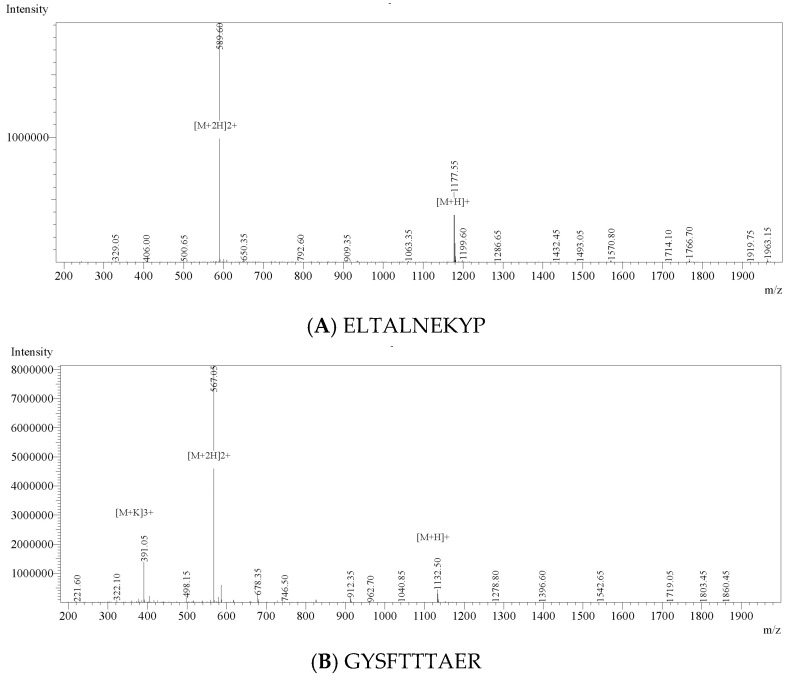

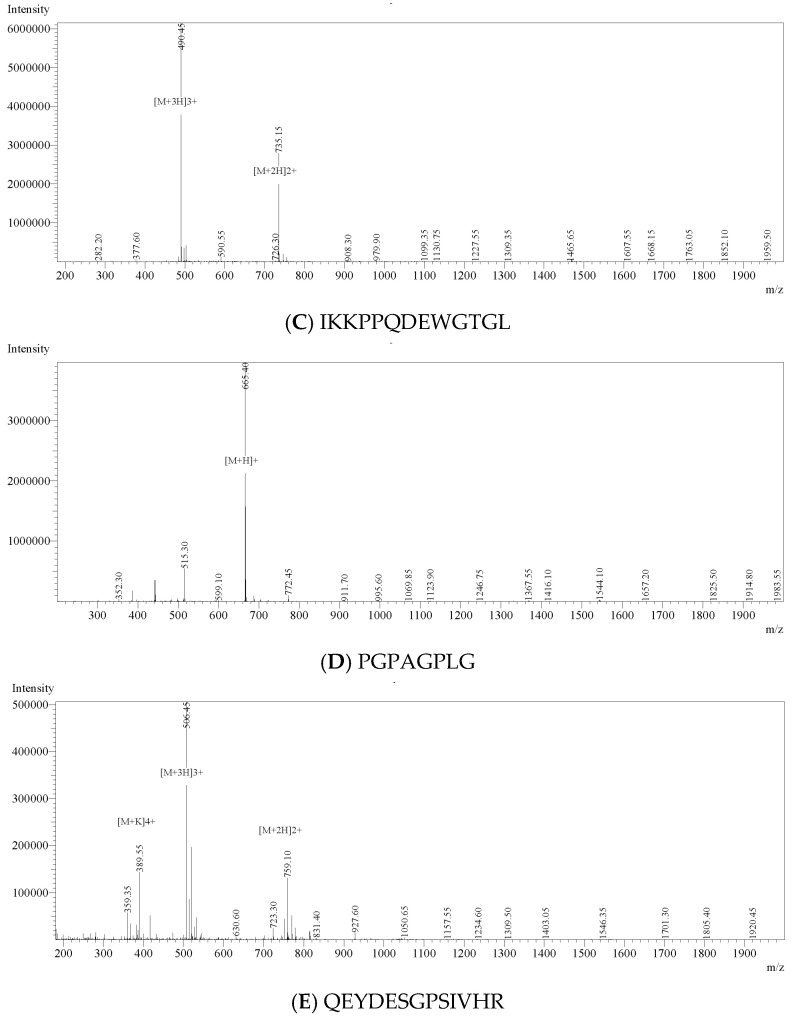
ESI-MS chromatogram of synthetic peptides.

LC-MS/MS profiling of AVZH resolved 18 previously unreported peptide sequences (Table 3), spanning 664.35–2230.06 Da. The anti-inflammatory potential of identified peptides was predicted using PeptideRanker, AIPpred, and PreAIP (Table S4). Among the 18 identified peptides, only PGPAGPLG, IKKPPQDEWGTGL, and QAGGLMNLFTNN scored > 0.5 in at least one predictor. Considering peptide abundance and consensus predictions, five peptides—GYSFTTTAER, IKKPPQDEWGTGL, QEYDESGPSIVHR, ELTALNEKYP, and PGPAGPLG—were selected for chemical synthesis and subsequent activity validation. These peptides were subsequently synthesised by solid-phase Fmoc chemistry; ESI-MS (Figure 4) and RP-HPLC (Figure S1, Table 4) confirmed >95% purity and exact molecular masses, satisfying the stringent criteria required for subsequent bioactivity assays.

### 3.5. Impact of Synthetic Anti-Inflammatory Peptides on Cell Viability and NO Secretion

**Figure 5:**
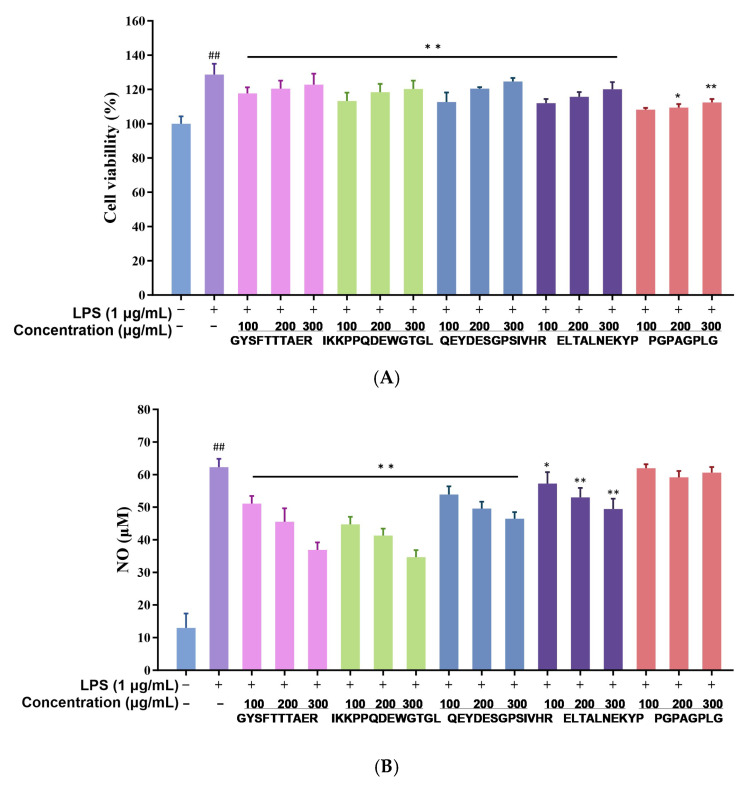
Effects of abalone visceral anti-inflammatory peptides on cell viability and NO secretion. (**A**) Effect of five kinds of abalone visceral peptides on the viability of RAW264.7 macrophages. (**B**) Effect of five kinds of abalone visceral peptides on the NO secretion of RAW264.7 macrophages. Values represent mean ± SD (*n* = 4); ## *p* < 0.01 versus control group; * *p* < 0.05, ** *p* < 0.01 vs. LPS control.

Across the concentration range 100–300 µg mL^−1^, none of the five synthetic peptides exerted cytotoxic effects on RAW264.7 macrophages; instead, they modestly enhanced cell proliferation, underscoring their excellent biocompatibility (Figure 5A). Consistent with the in silico predictions, GYSFTTTAER and IKKPPQDEWGTGL displayed the most potent suppression of LPS-induced NO release, with reductions of 29.8% and 34.7% at 300 µg mL^−1^, respectively (Figure 5B). Consequently, these two peptides were advanced to mechanistic studies.

### 3.6. Effects of Anti-Inflammatory Peptides on Cytokine Secretion and Transcriptional Expression in RAW264.7 Cells

**Figure 6:**
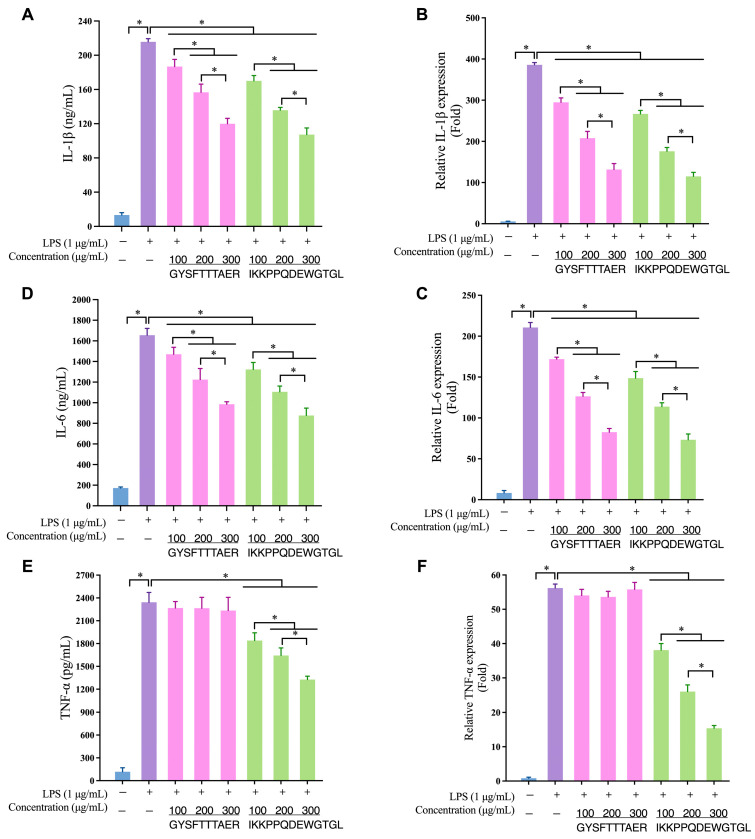
Effects of abalone visceral anti-inflammatory peptides on cytokine secretion and expression. ELISA analysis of GYSFTTTAER and IKKPPQDEWGTGL on (**A**) IL-1β, (**D**) IL-6 and (**E**) TNF-αsecretion by LPS-induced RAW264.7. qRT-PCR analysis of GYSFTTTAER and IKKPPQDEWGTGL on (**B**) IL-1β, (**C**) IL-6 and (**F**) TNF-αexpression in LPS-induced RAW264.7. Values represent mean ± SD (*n* = 4); * *p* < 0.05.

ELISA analyses revealed that GYSFTTTAER and IKKPPQDEWGTGL dose-dependently curbed the LPS-evoked release of IL-1β, IL-6 and TNF-α from RAW264.7 macrophages (Figure 6A–C). The most pronounced inhibition was achieved with IKKPPQDEWGTGL at 300 µg mL^−1^, which suppressed TNF-α, IL-1β and IL-6 secretion by 43.36%, 50.29% and 47.06%, respectively. Quantitative RT-PCR corroborated these findings at the mRNA level, demonstrating a concomitant down-regulation of the corresponding transcripts (Figure 6D–F). The tight concordance between protein and gene expression data confirms the robust anti-inflammatory efficacy of both peptides and underpins their mechanistic relevance.

### 3.7. Anti-Inflammatory Peptides Suppress MAPK Signaling Cascades in RAW264.7 Cells

**Figure 7:**
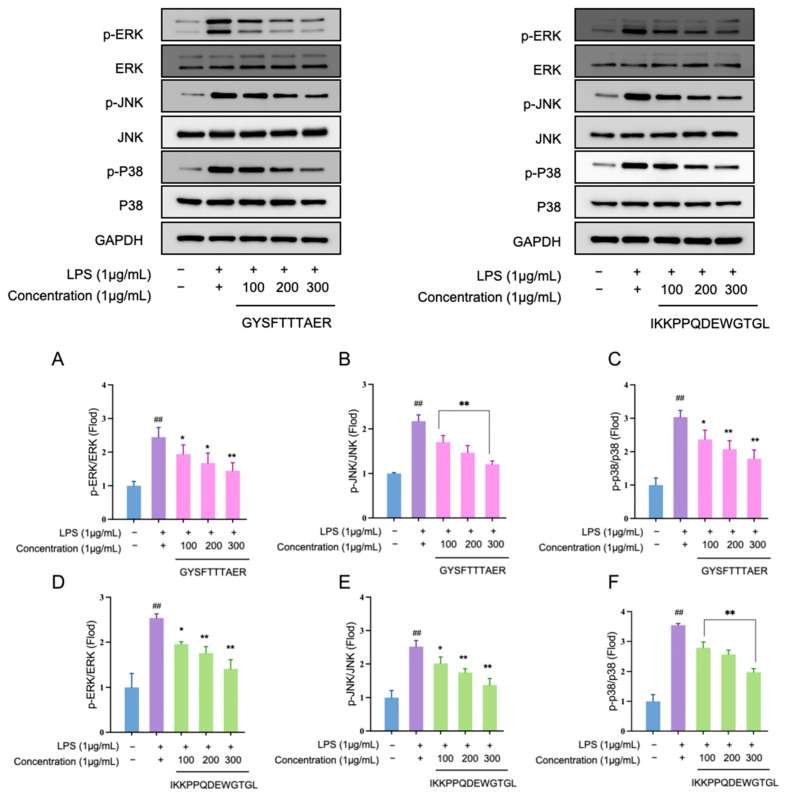
Effects of abalone visceral anti-inflammatory peptides GYSFTTTAER and IKKPPQDEWGTGL on the phosphorylation of the MAPK signaling pathway in LPS-induced RAW264.7 cells. (**A**,**D**) p-ERK expressions. (**B**,**E**) p-JNK expressions. (**C**,**F**) p-p38 expression. Values represent mean ± SD (*n* = 4); ## *p* < 0.01 compared to control group; * *p* < 0.05, ** *p* < 0.01.

Western blot analysis demonstrated that LPS stimulation markedly elevated the phosphorylation levels of ERK, JNK and p38; both GYSFTTTAER and IKKPPQDEWGTGL attenuated these increases in a concentration-dependent manner (Figure 7). At 300 µg mL^−1^, the peptides reduced p-ERK, p-JNK and p-p38 signals by 46–61% relative to the LPS control. These data indicate that the abalone visceral peptides exert their anti-inflammatory effects, at least in part, by interrupting MAPK activation, thereby preventing downstream transcription of pro-inflammatory mediators.

## 4. Discussion

Enzymatic hydrolysis serves as a cornerstone technology for recovering high-value bioactives from marine processing by-products [19]. In this study, we systematically evaluated the efficiency of four commercially available proteases in generating bioactive hydrolysates from abalone viscera, an underutilized resource rich in protein and other valuable components [20,21]. Our findings revealed that the hydrolysates were primarily composed of polypeptides, with the AVZH exhibiting the highest peptide content. DH varied significantly among the proteases, with AVYH showing the highest DH, likely due to its specific cleavage specificity, a phenomenon consistent with previous reports on marine protein hydrolysis [22]. Comprehensive amino acid profiling demonstrated that all hydrolysates were rich in glutamic acid, aspartic acid, and arginine, and were characterized by a marked enrichment of hydrophobic residues such as valine and leucine after enzymatic hydrolysis. This compositional shift is significant as hydrophobic residues are frequently associated with enhanced bioactivity, including antioxidant and anti-inflammatory potential [23,24].

The antioxidant profiles of the hydrolysates, assessed through multiple complementary in vitro assays, were both potent and enzyme-dependent. AVYH delivered the strongest ferric-reducing antioxidant power and the most potent DPPH• radical-scavenging activity, a potency notably superior to some other reported marine hydrolysates [25]. This superior activity was closely correlated with its highest proportion of low-molecular-weight peptides (<500 Da) and its abundant hydrophobic amino acids, which are known to facilitate electron or hydrogen donation [26]. Conversely, AVBH exhibited the greatest efficacy in scavenging •OH. These divergent antioxidant activities underscore the critical influence of enzyme selection on the resulting peptide profile and associated functionality.

Beyond antioxidant capacity, the hydrolysates were evaluated for their anti-inflammatory potential in an LPS-stimulated RAW264.7 macrophage model. AVZH emerged as the most potent inhibitor of NO production. This superior activity was closely linked to its specific molecular composition, namely a high proportion of peptides within the 500–3000 Da range and a significant enrichment of hydrophobic residues, a structure–activity relationship consistently observed for anti-inflammatory peptides from various marine sources [27,28]. Consequently, AVZH was selected for in-depth peptide identification. Subsequent LC-MS/MS profiling resolved 18 novel peptide sequences. The anti-inflammatory potential of these peptides is likely influenced by their specific amino acid composition, hydrophobicity, and sequence length [29]. Notably, the presence of hydrophobic residues (e.g., Leu, Ala) and positively charged residues (e.g., Arg, Lys), found at the N-terminus, C-terminus, or within the sequences of many identified peptides, is frequently associated with anti-inflammatory potential [30,31]. An integrated in silico screening workflow employing PeptideRanker [32], AIPpred [17], and PreAIP [18] was used to prioritize five candidate peptides (GYSFTTTAER, IKKPPQDEWGTGL, QEYDESGPSIVHR, ELTALNEKYP, and PGPAGPLG) for solid-phase synthesis and experimental validation.

The two synthesized peptides, GYSFTTTAER and IKKPPQDEWGTGL, significantly suppressed the hypersecretion of key pro-inflammatory cytokines. IKKPPQDEWGTGL demonstrated broad-spectrum, dose-dependent inhibition, significantly reducing the secretion of TNF-α, IL-1β, and IL-6. In contrast, GYSFTTTAER potently inhibited IL-1β and IL-6 secretion but did not significantly affect TNF-α, a selective cytokine suppression that has also been observed in anti-inflammatory peptides derived from sturgeon protein [33]. Quantitative RT-PCR analysis confirmed that these inhibitory effects occurred at the transcriptional level, with IKKPPQDEWGTGL markedly reducing TNF-α mRNA expression. This multi-cytokine suppression effectively disrupts the inflammatory cascade where these factors mutually potentiate each other’s expression [34], underscoring the potent and nuanced anti-inflammatory efficacy of the identified peptides.

Most significantly, we elucidated a novel molecular mechanism underlying the anti-inflammatory action of these abalone visceral peptides. The MAPK cascade, comprising ERK1/2, JNK, and p38, is a central signaling hub governing the transcription of inflammatory mediators [35]. For the first time, Western blot analysis revealed that both GYSFTTTAER and IKKPPQDEWGTGL attenuated the LPS-induced phosphorylation of ERK, JNK, and p38 in a concentration-dependent manner. At 300 μg mL^−1^, the peptides substantially reduced the phosphorylation levels of these kinases relative to the LPS control. This newly identified MAPK-mediated mechanism clearly differentiates our peptides from many other food-derived anti-inflammatory peptides that primarily act through the NF-κB pathway [36], highlighting a unique and specific mode of action that expands the repertoire of known mechanisms for marine bioactive peptides.

Notably, this study establishes an efficient enzymatic strategy for valorizing abalone viscera and systematically identifies two novel anti-inflammatory peptides, GYSFTTTAER and IKKPPQDEWGTGL. Their potent, multi-level suppression of inflammation—through the inhibition of pro-inflammatory cytokine expression and the novel blockade of the MAPK signaling axis—provides a robust mechanistic foundation for their potential application.

## 5. Conclusions

This study successfully established an efficient bioprocess for valorizing abalone viscera, an underutilized marine by-product. We systematically demonstrated that neutral protease hydrolysis yielded the most promising hydrolysate (AVZH), rich in low-molecular-weight peptides (500–3000 Da) and hydrophobic amino acids. For the first time, two novel peptides—GYSFTTTAER and IKKPPQDEWGTGL—were identified and functionally characterized. These peptides exhibited excellent cytocompatibility and potent, dose-dependent, anti-inflammatory activity in LPS-stimulated macrophages. Their efficacy was manifested through the significant suppression of key pro-inflammatory mediators (NO, IL-1β, IL-6, TNF-α) at both protein and gene expression levels. Crucially, we elucidated that the underlying molecular mechanism involves the inhibition of the MAPK signaling pathway, as evidenced by the reduced phosphorylation of ERK, JNK, and p38. These findings not only unveil the potential of abalone viscera as a sustainable source of bioactive peptides but also provide a solid mechanistic foundation for their application in the development of natural anti-inflammatory functional foods or nutraceuticals. Future research should focus on in vivo validation, structural optimization, and exploring the bioavailability and stability of these promising peptides in complex food systems.

## Figures and Tables

**Table 1:** Primer sequence.

Primer Name	Primer Sequence
GAPDH (mouse) F	ACCCTTAAGAGGGATGCTGC
GAPDH (mouse) R	CCCAATACGGCCAAATCCGT
TNF-α (mouse) F	TAGCCCACGTCGTAGCAAAC
TNF-α (mouse) R	ACAAGGTACAACCCATCGGC
IL-1β (mouse) F	TGCCACCTTTTGACAGTGATG
IL-1β (mouse) R	TGATGTGCTGCTGCGAGATT
IL-6 (mouse) F	CTCATTCTGCTCTGGAGCCC
IL-6 (mouse) R	CAACTGGATGGAAGTCTCTTGC

**Table 2:** Basic components of abalone viscera and its crude enzymatic hydrolysate.

Sample	Components %			
	Total Sugars	Total Protein	Moisture Content	Others
AVME	21.82 ± 1.46	49.41 ± 1.12	9.14 ± 1.61	19.63 ± 2.55
AVBE	20.33 ± 3.09	48.43 ± 1.57	7.78 ± 0.29	23.46 ± 2.86
AVZE	19.13 ± 1.82	50.98 ± 2.86	8.7 ± 1.14	21.19 ± 2.73
AVYE	19.84 ± 1.51	50.69 ± 1.18	11.56 ± 0.13	17.91 ± 0.52
Abalone Viscera powder	18.54 ± 2.96	45.09 ± 2.48	7.99 ± 0.98	26.38 ± 0.49

**Table 3:** Novel peptides identified from AVZH by LC-MS/MS.

Accession	Sequence	Length	Mass/Da	Intensity	Theoretical Iso-Electric Point	Net Charge at pH 7	Estimated Solubility
AAQ92368.1	QEYDESGPSIVHR	13	1515.70	44,101,000	4.65	−1.9	G
AAQ92368.1	GYSFTTTAER	10	1131.52	23,298,000	6	0	G
AYD59980.1	PTIIFEPGIDTHVLD	15	1665.86	20,254,000	4.02	−2.9	P
QOD61783.1	ELTALNEKYP	10	1176.60	20,189,000	4.53	−1	G
AAQ92368.1	SYELPDGQVITIGNER	16	1789.88	18,912,000	4.14	−2	G
ADK60915.1	IKKPPQDEWGTGL	13	1467.77	12,707,000	6.07	0	G
AAQ92368.1	QEYDESGPSIVHR	13	1515.70	12,408,000	4.65	−1.9	G
APB93432.1	KHTLPDLPYDY	11	1360.67	11,330,000	5.21	−0.9	G
ADK60915.1	LKRVGPGLGEYQ	12	1315.72	10,870,000	8.59	1	G
AAQ92368.1	DLYANTVLSGGTTMYPGIADR	21	2230.06	8,836,800	4.21	−1	P
AYD59980.1	NHGSQIGIPY	10	1084.53	8,414,300	6.74	0.1	P
ADK60915.1	VGPGLGEYQFDHETLS	16	1747.81	3,786,000	4.13	−2.9	G
AYL88760.1	PGPAGPLG	8	664.35	3,689,900	5.96	0	P
AYD59980.1	PTIIFEPGIDTHV	13	1437.75	2,781,800	4.35	−1.9	P
QOD61838.1	ITNNQVQVITQAP	13	1428.70	2,387,900	5.52	0	P
AAQ92368.1	DLTDYLMK	8	997.48	1,485,200	4.21	−1	G
UJF23575.1	QAGGLMNLFTNN	12	1295.58	1,485,200	5.52	0	P
GDY27137.1	NNGKVIVEVGQP	12	1254.65	1,485,200	6	0	G

Note: G denotes good water solubility, P denotes poor water solubility.

**Table 4:** Synthetic peptide chromatographic analysis statistical.

Peptide Sequence	Peak #	Ret. Time (min)	Area %
QEYDESGPSIVHR	2	10.295	95.076
GYSFTTTAER	2	12.295	98.192
ELTALNEKYP	4	11.231	98.439
IKKPPQDEWGTGL	3	9.770	95.282
PGPAGPLG	1	9.436	99.119

## Data Availability

The original contributions presented in the study are included in the article/Supplementary Material, further inquiries can be directed to the corresponding author.

## Supplementary Materials

The following supporting information can be downloaded at: https://www.mdpi.com/article/10.3390/foods14223811/s1.

## Abbreviations

Abbreviation	Full Form
ACE	Angiotensin-I-converting Enzyme
ANOVA	Analysis of Variance
AV	Abalone Viscera
AVBH	Bromelain Hydrolysate of Abalone Viscera
AVMH	Papain Hydrolysate of Abalone Viscera
AVYH	Trypsin Hydrolysate of Abalone Viscera
AVZH	Neutral-protease Hydrolysate of Abalone Viscera
DPPH	2,2-Diphenyl-1-picrylhydrazyl
ERK	Extracellular Signal-Regulated Kinase
ESI-MS	Electrospray Ionization Mass Spectrometry
FBS	Fetal Bovine Serum
GAPDH	Glyceraldehyde-3-Phosphate Dehydrogenase
HP-GPC	High-Performance Gel Permeation Chromatography
IL-1β	Interleukin-1 Beta
IL-6	Interleukin-6
JNK	C-Jun N-terminal Kinase
LC-MS/MS	Liquid Chromatography with Tandem Mass Spectrometry
LPS	Lipopolysaccharide
MAPK	Mitogen-activated Protein Kinase
NO	Nitric Oxide
p-ERK	Phosphorylated Extracellular Signal-Regulated Kinase
p-JNK	Phosphorylated C-Jun N-terminal Kinase
p-p38	Phosphorylated p38
p38	p38 Mitogen-Activated Protein Kinase
RP-HPLC	Reversed-Phase High-Performance Liquid Chromatography
TNF-α	Tumour Necrosis Factor-alpha
