# Supplementary material for: Purification and Characterization of Anti-Inflammatory Peptide Fractions from Enzymatic Hydrolysate of Abalone Viscera
Source: Foods. 2025 Nov 7;14(22):3811. doi: 10.3390/foods14223811 (PMC12651024; doi:10.3390/foods14223811)
Supplement: Supplementary file 1 [file foods-14-03811-s001.zip › foods-3958650-supplementary.pdf]

Table S1. Basic composition of abalone offal and its enzymatic digests

sample	contents	
	peptide（%）	DH（%）
AVMH	58.50±2.32b	11.43±0.59ab
AVBH	54.14±1.79b	10.78±1.64c
AVZH	68.38±3.87a	12.44±0.76ab
AVYH	64.69±2.28a	14.35±1.39a
Dried abalone offal powder solution	2.46±0.38c	-

Table S2 Amino acid composition of abalone viscera and its enzymatic hydrolysates

Amino acid	Quality Ingredients（%）				
	AV	AVZH	AVBH	AVYH	AVMH
Aspartate	11	9	10	10	9
Glutamate	13	16	16	10	12
Cystine	-	-	-	-	-
Serine	6	4	5	3	3
Glycine	6	3	7	4	3
Histidine	2	2	3	2	3
Arginine	14	8	5	7	17
Threonine	5	5	6	5	3
Alanine	7	10	6	9	9
Proline	7	10	8	8	15
Tyrosine	2	5	2	4	2
Valine	6	7	7	11	8
Methionine	2	2	2	2	1
Isoleucine	6	5	6	8	5
Leucine	7	8	7	9	6
Phenylalanine	1	3	4	4	2
Lysine	5	3	6	4	2
Total	100	100	100	100	100
Hydrophobic amino acids	38	50	42	55	48

Table S3 Molecular weight distribution of four types of abalone visceral enzymatic hydrolysates

MW（Da）	Relative content（%）			
	AVMH	AVZH	AVBH	AVYH
>3000	2.29	2.53	7.31	2.59
3000-500	67.9	73.46	66.99	51.63
<500	29.81	24.01	25.71	45.79

Table S4 Prediction of potential activity of 18 anti-inflammatory peptides

**Sequence**	PeptideRanker	PreAIP	AIPpred
QEYDESGPSIVHR	0.214575	Medium Confidence AIP	AIP
GYSFTTTAER	0.170181	Medium Confidence AIP	AIP
ELTALNEKYP	0.109014	Low Confidence AIP	AIP
PTIIFEPGIDTHVLD	0.209295	Medium Confidence AIP	AIP
SYELPDGQVITIGNER	0.206175	High Confidence AIP	AIP
IKKPPQDEWGTGL	0.750647	High Confidence AIP	AIP
QEYDESGPSIVHR	0.214575	Medium Confidence AIP	AIP
KHTLPDLPYDY	0.193634	Low Confidence AIP	non
LKRVGPGLGEYQ	0.157741	Medium Confidence AIP	AIP
DLYANTVLSGGTTMYPGIADR	0.089817	High Confidence AIP	AIP
NHGSQIGIPY	0.311116	Medium Confidence AIP	AIP
VGPGLGEYQFDHETLS	0.259748	Medium Confidence AIP	AIP
PGPAGPLG	0.895354	negative AIP	AIP
PTIIFEPGIDTHV	0.196097	High Confidence AIP	AIP
ITNNQVQVITQAP	0.041926	Medium Confidence AIP	AIP
DLTDYLMK	0.429737	Low Confidence AIP	AIP
QAGGLMNLFTNN	0.524976	Medium Confidence AIP	non
NNGKVIVEVGQP	0.09619	High Confidence AIP	AIP

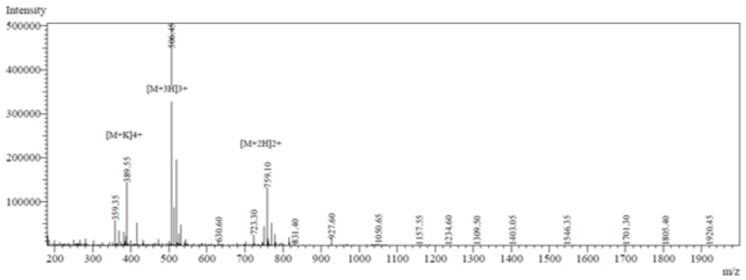

（a）QEYDESGPSIVHR

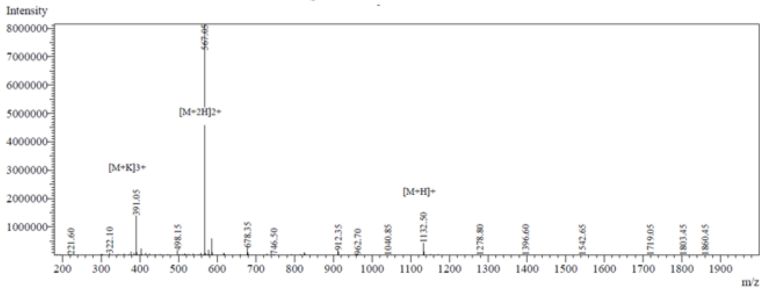

（b）GYSFTTTAER

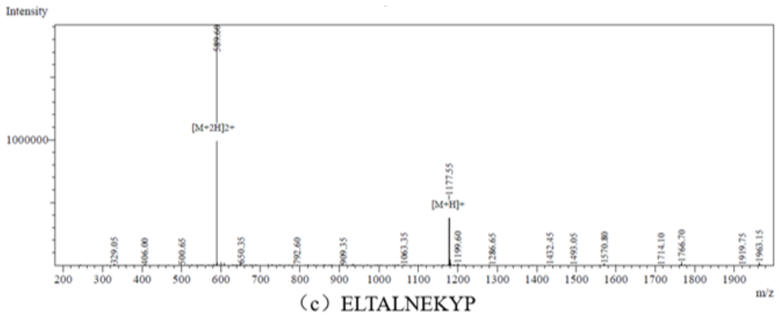

（c）ELTALNEKYP

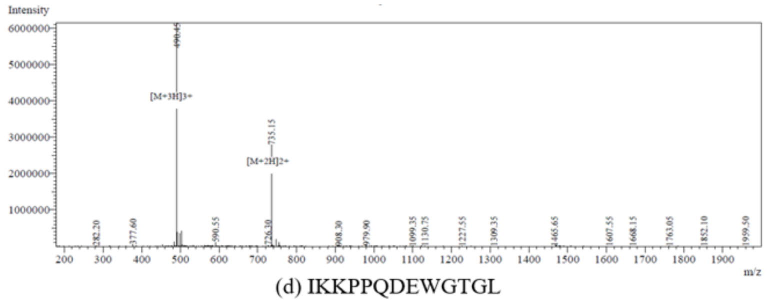

(d) IKKPPQDEWGTGL

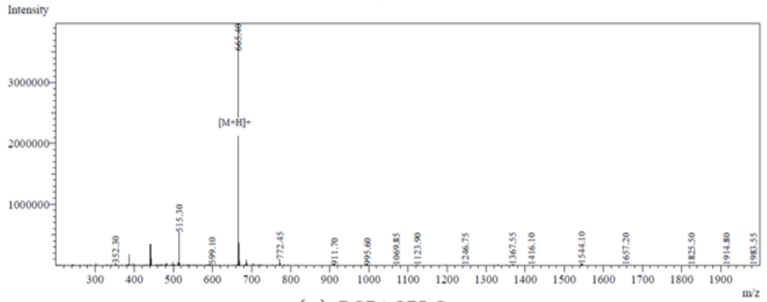

（e）PGPAGPLG

Figure S1 Mass spectrum of Synthetic peptide
